# Rotating Machinery Diagnosing in Non-Stationary Conditions with Empirical Mode Decomposition-Based Wavelet Leaders Multifractal Spectra

**DOI:** 10.3390/s21227677

**Published:** 2021-11-18

**Authors:** Iwona Komorska, Andrzej Puchalski

**Affiliations:** Department of Mechanical Engineering, Kazimierz Pulaski University of Technology and Humanities in Radom, Malczewskiego 29, 26-600 Radom, Poland; andrzej.puchalski@uthrad.pl

**Keywords:** wavelet leaders, multifractal spectrum, rotating machines, fault diagnostics

## Abstract

Diagnosing the condition of rotating machines by non-invasive methods is based on the analysis of dynamic signals from sensors mounted on the machine—such as vibration, velocity, or acceleration sensors; torque meters; force sensors; pressure sensors; etc. The article presents a new method combining the empirical mode decomposition algorithm with wavelet leader multifractal formalism applied to diagnosing damages of rotating machines in non-stationary conditions. The development of damage causes an increase in the level of multifractality of the signal. The multifractal spectrum obtained as a result of the algorithm changes its shape. Diagnosis is based on the classification of the features of this spectrum. The method is effective in relation to faults causing impulse responses in the dynamic signal registered by the sensors. The method has been illustrated with examples of vibration signals of rotating machines recorded on a laboratory stand, as well as on real objects.

## 1. Introduction

When properly processed, dynamic signals recorded during the operation of rotating machines are a valuable source of information about the condition of machines. Algorithms are still being developed, which will quickly, unequivocally, and automatically determine the operational state of the machine. One of the most commonly used methods in diagnostics are vibration sensors due to the ease of assembly, the possibility of obtaining information without disassembling the machine, and the wealth of information obtained about the condition of the machine in various frequency ranges. In the case of machine operation with constant speed and load, the most effective method of analysis is the frequency spectrum [1]. However, due to the fluctuating rotational speed of the machine, often due to a variable load, the frequency spectrum becomes blurred. The real challenge is to diagnose machines in non-stationary conditions. In order to avoid resampling in the analysis of dynamic signals, time–frequency methods are used [2]. The authors present a systematic review of over 20 time–frequency methods used to detect machine damage. One of the simplest is the short-time Fourier transform STFT [3]. The improved version of the spectrogram is the Wigner–Ville analysis, the use of which in diagnosing damage to gears is described in [4,5]. The use of wavelet analysis in diagnosing damage to rotating machines can be found, among others in [6,7]. Other papers [8,9,10] present a method of detecting and diagnosing gear damage on the basis of a reference model based on the signal averaging technique. The proposed algorithm first establishes an autoregressive (AR) model of the gear vibration signal in its base state, and then diagnoses the state based on the residual signal. The use of the synchronous method (order-tracking analysis) allows to synchronize the diagnostic signal with the rotation of the diagnosed machine, as well as to eliminate the influence of background noise from other sources [11,12]. The article [13] proposes a method of informative selection of frequency bands. It utilizes the approach of non-negative matrix factorization applied to time–frequency signal representation. For the analysis of non-stationary signals, the empirical mode decomposition EMD algorithm is also used, which decomposes the signal into modes in an empirical manner [14,15]. Since the classic method can mix two or three modes, various modifications of the EMD method are developed [16,17,18].

In recent years, more and more often, efforts are being made to automate the diagnosis process, and qualified diagnosticians are to be replaced by diagnostic algorithms. So-called data-driven statistical methods can also be successfully used in diagnosing machines in non-stationary conditions. Determining the characteristics of a dynamic signal and its classification is carried out by machine learning algorithms [19,20,21,22]. More and more papers are devoted to the diagnostics of machines with the methods of deep learning [23,24,25,26,27,28,29]. Methods that allow detecting failures at an early stage, especially in non-stationary conditions that often occur in real objects, are still being searched and developed.

The fractal theory has unique advantages in dealing with transient, nonlinear, and non-stationary signals. It is a new trend in solving practical problems involving the study of short-term signals in a transient process with strong nonlinearity, based on the fractal theory in order to diagnose damage to mechanical equipment. The impact of damage to the outer race of a rolling bearing on the width of the multifractal spectrum is presented in [30]. Detrended fluctuation analysis MF-DFA is a commonly used algorithm for multifractal analysis. The method is often used to diagnose damage to rolling bearings [31,32,33] and gears [34,35,36]. In [37], the MF-DFA algorithm was used for the analysis of frictional vibrations, where the ensemble empirical mode decomposition EEMD was used to denoise the signal. The adaptive MF-DFA algorithm is presented in [38]. The disadvantage of the MF-DFA algorithm is its sensitivity to analysis parameters. These disadvantages are not present in the method of wavelet leaders (WLMF). The wavelet leaders algorithm was used to analyze the images [39] and diagnose damage to rotating machines [40].

The article proposes a novel EMD-WLMF method consisting in the multifractal analysis of the first empirical component (IMF1) of the vibration signal using WLMF algorithm. As a result, a multifractal spectrum is obtained, the parameters of which are used to distinguish the state of the machine. Since the tested damage causes impulse disturbances of the vibration signal, the signal is first filtered out using the empirical decomposition of the signal EMD. Only the first intrinsic mode function (IMF1) containing high-frequency information is subject to multifractal analysis. The method has been illustrated with examples of signals recorded on a laboratory stand as well as on real objects. The presented examples, especially the analysis of signals recorded in the vehicle, show that the combination of EMD and WLMF methods allows the detection of damage at an early stage in conditions unfavorable to diagnosis—i.e., at floating rotational speed and load.

The article is organized as follows. Section 2 describes the methods of signal analysis used in the study, i.e., the WLMF algorithm, as well as determining the first intrinsic mode function (IMF1) using the EMD method. In Section 3, the described algorithm for fault diagnosis was used on the basis of signals recorded on a laboratory stand and on real objects under operating conditions. The discussion is presented in Section 4.

## 2. Materials and Methods

### 2.1. Multifractal Formalism

Multifractal analysis, which is based on estimated signal scaling exponents, is a popular statistical tool for studying time series. The mathematical formalism is based on the increments of their values, the measure of which are the pointwise Holder exponents *h* of the time function *x*(*t*) at the point *t*_0_, determined by the supremum of all exponents satisfying, for the constant *C* > 0, the condition

(1) $$\left|x\left(t\right)-P_{n}\left(t-t_{0}\right)\right|\leq C{\left|t-t_{0}\right|}^{h}$$

where $P_{n}\left(t-t_{0}\right)$ is a polynomial of the order *n* < *h.* The pointwise Holder exponent describes the regularity of the function. The greater the local regularity of the time series, the higher the singularity exponent. The multifractal spectrum *D*(*h*) represents the histogram of the Holder exponents and determines the fractal dimensions of the subsets of singularities with a given exponent value [41,42,43].

In the time–frequency approach to signal analysis, the WTMM (wavelet transform modulus maxima) method was initially used, which is based on a continuous wavelet transform (CWT). It consists in determining the chains of local, the structure function and its scaling exponents, as well as the Legendre transform. Practical implementations of such an algorithm showed a number of faults that make it impossible to conduct research for some types of real signals [44].

Another approach to the problem of estimating local scaling exponents as a method of studying the regularity of time series and their multifractality is related to the multifractal detrended fluctuation analysis (MF-DFA) method. MF-DFA enables to study the observed signals in terms of their multifractality, provides a more stable approach to multifractal formalism than the WTMM method [31,36,45,46,47].

The formalism in the time–frequency domain used in the paper allows for the estimation of multifractal parameters using wavelet leaders (WLMF), which are representatives of local Holder exponents of the signal. In the case of wavelet coefficients centered around zero values, it is difficult to guarantee numerical stability. This problem does not arise in the case of the WLMF method, based on the wavelet coefficients obtained as a result of the discrete wavelet transform (DWT). The next steps of the algorithm include the selection of coefficients called wavelet leaders, determination of the structure function with scaling exponents, and the multifractal spectrum. The algorithm shows low computational costs, numerical stability, and high versatility in terms of real signals [39,40].

The scaling exponents of the structural function do not depend on the selection of the wavelet, provided that the number of zero wavelet moments is two times greater than the largest exponent of the signal holder. The mother wavelet must be orthogonal or biorthogonal. In the work, the Daubechies (db2) wavelet mother was arbitrarily selected.

For the coefficients (2) of the discrete wavelet transform (DWT) of the function *x*(*t*) and the basic wavelet with a compact support *ψ*_0_ (*t*)

(2) $$d_{x}\left(j,k\right)=\int_{R}^{}x\left(t\right)2^{-j}\psi_{0}\left(2^{-j}t-k\right)dt$$

wavelet leaders (3), for the set of the largest coefficients $d_{x}\left(j^{\prime },k^{\prime }\right)\equiv d_{\lambda^{\prime }}$ in the vicinity of 3*λ*, are defined by the dependence on any scale

(3) $$L_{x}\left(j,k\right)=sup_{\lambda^{\prime }\in 3\lambda }\left|d_{\lambda^{\prime }}\right|$$

where j,k are integers and $3\lambda ∶=3\lambda_{j,k}=\lambda_{j,k-1}\cup^{\;}\lambda_{j,k}\cup^{\;}\lambda_{j,k+1}$ and $\lambda ∶=\lambda_{j,k}=\left[k2^{j},\left(k+1\right)2^{j}\right]$. $L_{x}\left(j,k\right)$ consists of the largest wavelet coefficient $d_{x}\left(j^{\prime },k^{\prime }\right)$ computed at all finer scales $2^{j^{\prime }}\leq 2^{j}$ within a narrow time neighborhood $\left(k-1\right)\cdot 2^{j}\leq 2^{j^{\prime }}k^{\prime }<\left(k+2\right)\cdot 2^{j}$.

It can be shown that the Holder exponents are the scaling exponents of wavelet leaders: $L_{x}\left(j,k\right)\sim 2^{jh}$. Moreover, the structure function (4) defined for wavelet leaders is described by the power dependence, the exponent of which is the multifractal scaling exponent ζ(q):R→R.

(4) $$Z_{L}\left(q,j\right)=\frac{1}{n_{j}}\sum_{k=1}^{n_{j}}L_{x}{\left(j,k\right)}^{q}=EL_{x}{\left(j,k\right)}^{q}\sim 2^{j\zeta \left(q\right)}$$

where q is the order of the structure function and $n_{j}$ is the number of intervals of the multiresolution analysis.

The function obtained by the Legendre transformation of the multifractal scaling exponent ζ(q), under mild conditions of signal regularity, is the upper limit for the multifractal spectrum (5) of the tested signal

(5) $$D\left(h\right)\leq min_{q\neq 0}\left[1+qh-\zeta \left(q\right)\right]$$

The description of dynamic properties of systems is successfully carried out on the basis of the parameters of multifractal spectra of representative time series.

The following were selected as parameters of the multifractal spectrum D(h) related to singularities h representing the local scaling of the measure in different places of the time series:

- multifractality level, representing the heterogeneity of the signal under study, $∆=h_{max}-h_{min}$, where $h_{max}$ and $h_{min}$ are the singularities corresponding to the largest and the smallest fluctuations in the time series (observed signal);
- span of dimensions of subsets of singularities $∆D=D\left(h_{max}\right)-D\left(h_{min}\right)$;
- the singularity with the greatest dimension, which is the most common singularity of the time series $\left\{h_{0}:D(h_{0)}=\max D\left(h\right)\right\}$.

**Figure 1:**
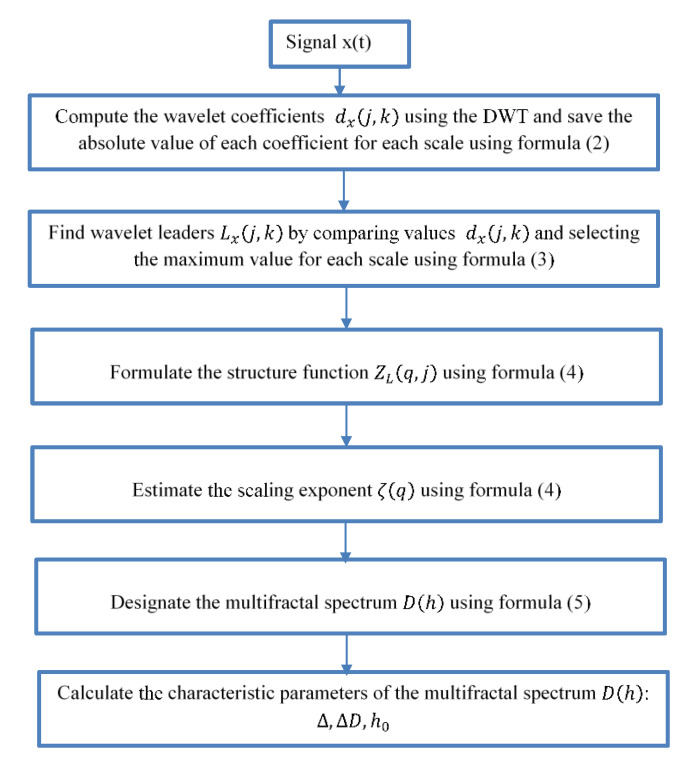
The method scheme.

The procedure of the method is shown in Figure 1.

**Figure 2:**
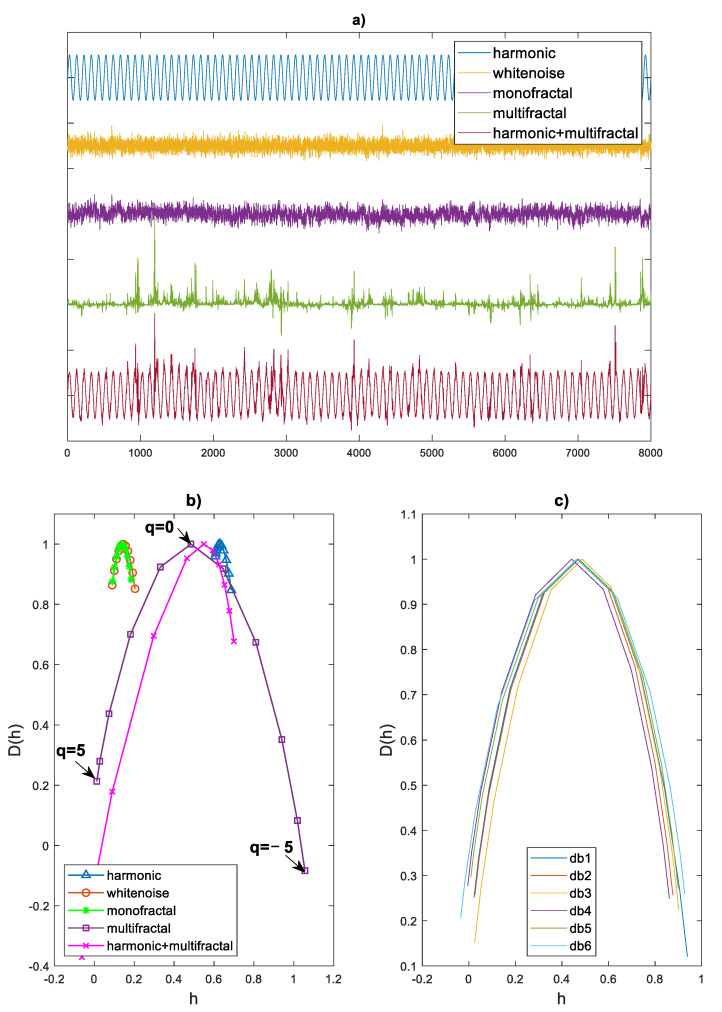
Multifractal analysis: (**a**) time series, (**b**) multifractal spectra with characteristic points, (**c**) multifractal spectra for Daubechies mother wavelets of orders 1 to 6.

The effect of the algorithm is illustrated in Figure 2 for a harmonic signal and various types of noise, examined in terms of multifractality. Figure 2a shows sample waveforms of signals, Figure 2b shows their multifractal spectra with the characteristic points. The harmonic signal, three types of random signals varying in the level of multifractality, and the sinusoidal signal disturbed by the multifractal signal were analyzed. The level of multifractality can be measured by the width of the multifractal spectrum. Thus, both the harmonic signal and the white noise cannot be treated as multifractal signals. Multifractal signals are characterized by impulse disturbances, such as harmonic signals with noise.

Figure 2b shows the characteristic points of the multifractal spectrum, which were used to define the characteristics of the spectrum:

- Spectrum width

(6) width=∆=h(q=−5)−h(q=5)

- Spectrum asymmetry

(7) asymmetry=∆D=D(q=−5)−D(q=5)

- The singularity exponent with the greatest fractal dimension

(8) $$shift=h_{0}=h\left(q=0\right)$$

Figure 2c shows the multifractal spectra determined when selecting the Daubechies mother wavelet of orders 1 to 6. As the selection of the wavelet order has little effect on the parameters of the spectrum, the Daubechies wavelet of order 2 (‘db2’) was arbitrarily selected. The selection of Daubechies wavelets of different orders should yield consistent results when used repeatedly.

### 2.2. Empirical Mode Decomposition

The EMD is an iterative numerical approximation algorithm designed to extract intristic mode functions IMFs from signals adaptively by cubic spline interpolation according to the local characteristic time-scale. It decomposes a signal into IMFs via iterative sifting.

Any signal can be approximated by a superposition of a series of IMFs as written by

(9) $$x\left(t\right)=\sum_{i=1}^{n}c_{i}\left(t\right)+r_{n}\left(t\right),$$

where $c_{i}\left(t\right)$ is the i-th IMF, and $r_{n}\left(t\right)$ is the residual signal which represents the slowly varying or constant trend of the signal.

Applying the Hilbert transform [14] to each IMF in Equation (9), we can construct the corresponding analytic signal. Then we can compute the instantaneous frequency via the derivative of instantaneous phase relative to time. By expressing the analytic signals in polar coordinate form, and taking the real part, we can obtain the Hilbert amplitude spectrum

(10) $$TFR_{x}\left(t,f\right)=Re\overset{n}{\underset{i=1}{\sum }}\left\{a_{i}\left(t\right)exp\left|j\int^{\;}2\pi f_{i}\left(t\right)dt\right|\right\}$$

and Hilbert energy spectrum

(11) $$TFR_{x}\left(t,f\right)=\overset{n}{\underset{i=1}{\sum }}a_{i}{}^{2}\left(t\right)\delta \left[f-f_{i}\left(t\right)\right]$$

where δ(·) is the Dirac delta function.

The EMD is the core of the Hilbert–Huang transform. For a real signal x(t), the EMD procedure is as follows.

1. Initialize parameters: Set iteration index *i* = 1, residual signal $r_{0}\left(t\right)=x\left(t\right).$
2. Extract the *i*-th IMF:
  a. Let *j* = 0, and $h_{ij}\left(t\right)=h_{i-1}\left(t\right)$.
  b. Find the local minima and the local maxima of $h_{ij}\left(t\right).$
  c. Interpolate the local minima and the local maxima with cubic spline to construct the lower and the upper envelopes of $h_{ij}\left(t\right).$
  d. Compute the instantaneous mean of the lower and upper envelopes $m_{ij}\left(t\right)$
  e. Let $h_{ij}\left(t\right)=h_{ij}\left(t\right)-m_{ij}\left(t\right)$.
  f. If $h_{ij}\left(t\right)$ satisfies the stop criteria for IMF sifting, then set the *i*-th IMF $c_{i}\left(t\right)=h_{ij}\left(t\right)$. Otherwise, let *j* = *j* + 1, return to step 2b.
3. Let $r_{i}\left(t\right)=r_{i-1}\left(t\right)-c_{i}\left(t\right)$.
4. If $r_{i}\left(t\right)\;$satisfies the stop criteria for EMD, then set $r_{i}\left(t\right)\;$as the residue, and terminate the EMD process. Otherwise, let i=i+1, return to Step 2.

To illustrate the method, a simulated signal was used, which shows the vibrations of a gear transmission operating at a variable rotational speed [9].

(12) $$x\left(t\right)=\overset{M}{\underset{m=0}{\sum }}A_{m}\left[1+{\tilde{a}}_{m}\left(t\right)\right]\cos\left\{2\pi f_{m}\left(t\right)+\beta_{m}+{\tilde{\theta }}_{m}\left(t\right)\right\}+z\left(t\right)+\phi \left(t\right)$$

where *m* (0,1,…,*M*) is the meshing harmonic number, $A_{m}$ the amplitude at the *m*-th harmonic frequency $f_{m}$ (i.e., $f_{m}=m\cdot N\cdot f_{s}\left(t\right)$, where *N* is the tooth number and $f_{s}$ is the shaft rotation frequency), t time vector (with sampling time dt), $\beta_{m}$ the initial phase, z(t) the impact-induced resonant vibration, ${\tilde{a}}_{m}\left(t\right)$ and ${\tilde{\theta }}_{m}\left(t\right)$ are the modified amplitude and phase modulation functions at the *m*-th harmonic, respectively, ϕ(t)  the white noise.

The following model parameters were adopted for the simulation: *M* = 2, $A_{1}=5$, $A_{2}=1.25$, *N* = 10, $\beta_{m}=0$, *dt* = 0.01 s; ${\tilde{a}}_{1}\left(t\right)=0.1\sin\left(2\pi f_{s}\cdot t\right)$, ${\tilde{a}}_{2}\left(t\right)=0.025\sin\left(4\pi f_{s}\cdot t\right)$; ${\tilde{\theta }}_{1}\left(t\right)=4.7\sin\left(2\pi f_{s}\cdot t\right)$, ${\tilde{\theta }}_{2}\left(t\right)=1.2\sin\left(4\pi f_{s}\cdot t\right)$. $f_{s}\left(t\right)$ is a random walk around the value 1. z(t) is described as a convolution of the resonant response to impact during one rotation and the pulse train z(t)=h(t)∗g(t), where h(t)=30sin(40π·t)·exp(−10t) and g(t) is the sequence of pulses occurring once per revolution.

Signal-to-noise-ratio, SNR, is calculated as

(13) $$SNR=10\;\log_{10}\frac{\sum_{i=1}^{n}{\left(s_{i}\right)}^{2}}{\sum_{i=1}^{n}{\left(w_{i}\right)}^{2}}$$

where $s_{i}$ is *i*-th amplitude of the signal and $w_{i}$ *i*-th amplitude of the noise. For a simulated signal, SNR = 4.6 dB. The main components of signal power are visible impacts.

**Figure 3:**
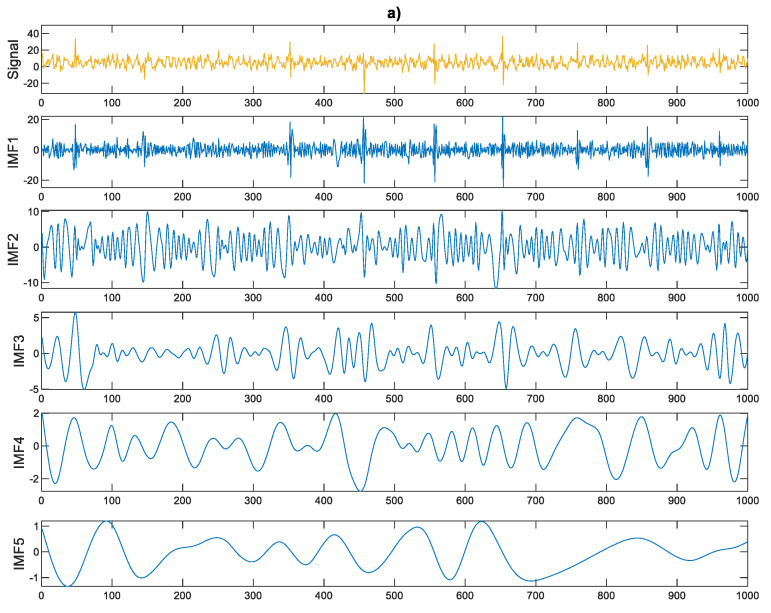

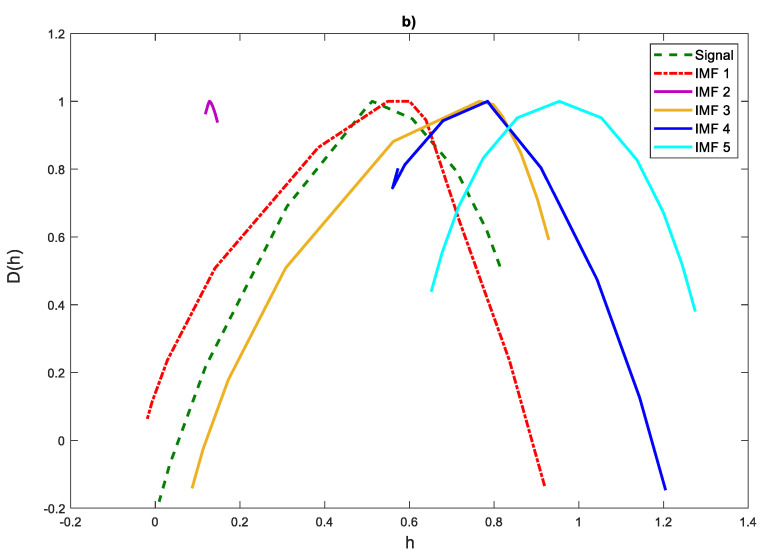
Simulated signal and five intristic mode functions (**a**) and their multifractal spectra (**b**).

The signal includes both quasi-harmonic components and impulse responses related to the damage of the gear. The signal presented by the Formula (12) is shown in Figure 3a, together with the following intristic mode functions from 1 to 5, which are the result of decomposition. Figure 3b shows the multifractal spectra for waveforms illustrated in Figure 3a.

The first empirical mode reveals the high-frequency impacts. This high-frequency mode increases in energy as the wear or damage progresses. The next modes show the resonance in the vibration signal and rotating frequency. The first intristic mode function will be used for further analysis. The spectrum for IMF1 is broader and more regular than the spectrum of the raw signal and the other IMF, while the IMF2 is a monofractal.

### 2.3. EMD-WLMF Method

**Figure 4:**
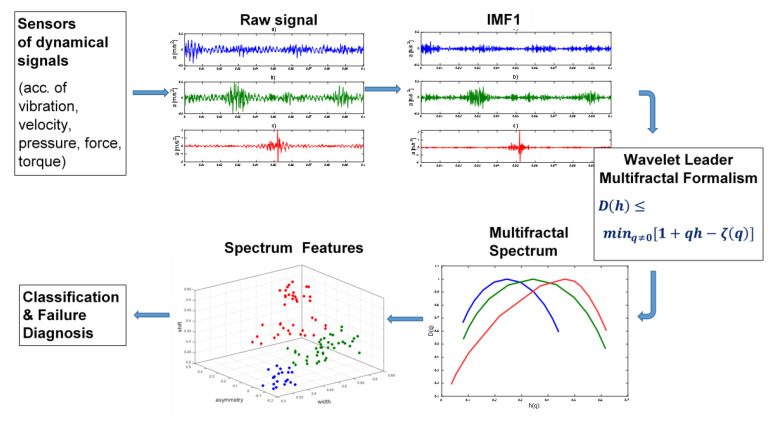
Diagram of the EMD- WLMF method.

Figure 4 shows a diagram of the EMD-WLMF method. From a dynamic signal using EMD analysis is extracting the first IMF. Then, using the WLMF algorithm, the signal parameters *h* and *D*(*h*) are determined, on the basis of which a multifractal spectrum can be drawn. Based on the characteristics of this spectrum, the operational state of the rotating machine is classified.

## 3. Application of the EMD-WLMF Algorithm in Diagnostics of Rotating Machines

The method presented in Section 2.3 was tested on the vibration signals recorded for the gear transmission on a laboratory stand and on a real object, which is a passenger car while driving.

### 3.1. Gear Transmission Vibration Signal Analysis on a Laboratory Stand

**Figure 5:**
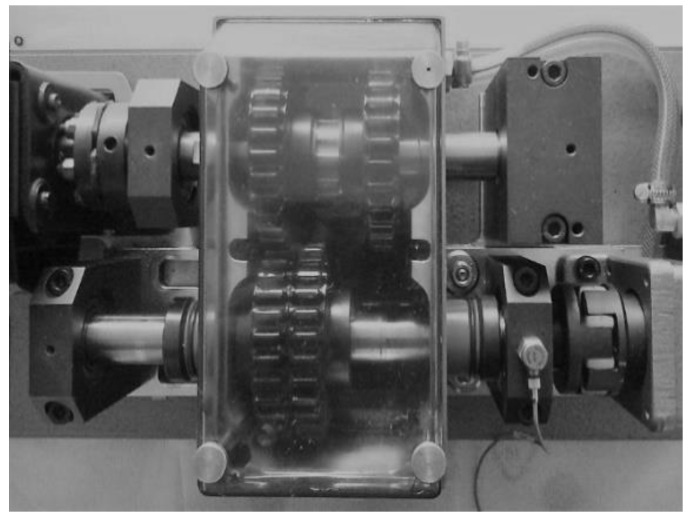
The test stand [48].

Measurements were carried out on a demonstration stand (Figure 5). The influence of assembly errors and gear teeth wear on vibrations was investigated. The electric motor enables speed control in the range of 100–3000 rpm (without load). The load is pressure regulated with an overflow valve up to 5 MPa. Vibration acceleration was measured with an accelerometer bolted to the gear bearing housing in the vertical direction. The optimal backlash was set to 0.1 mm.

Compared to previous papers [48], the experiment was extended with additional intermediate operating states and the analysis method was modified.

Measurements were carried out for the following five states:

- fault-free (new gears, the optimal backlash, parallel shaft location);
- new gears and misalignment by an angle up to 1/3° (two cases);
- new gears and increased backlash +0.1 mm up to +0.3 mm (three cases);
- worn teeth (three cases);
- worn teeth and increased backlash +0.3 mm (two cases).

Vibration acceleration signals were recorded for a rotational speed of about 1050 rpm and a load of 12%—pressure 0.6 MPa. Each sample included a time series with a length of *n* = 10,000, recorded at a sampling frequency of 10 kHz.

**Figure 6:**
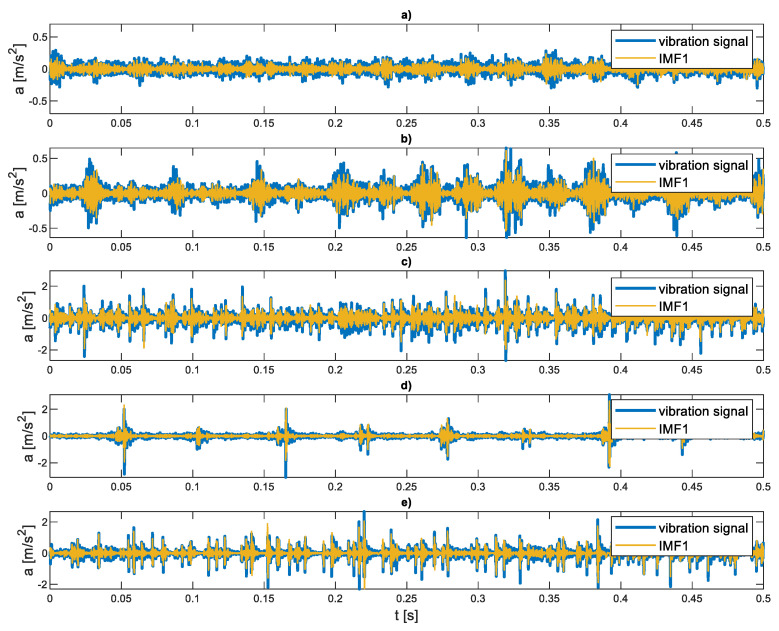
Time series of the vibration signal in various operating states (**a**) fault-free state; (**b**) misalignment 1/3°; (**c**) increased backlash 0.2 mm; (**d**) worn teeth second stage; (**e**) worn teeth second stage, and increased backlash 0.2 mm.

The representative waveforms of the vibration acceleration signal for each of the five states are shown in Figure 6. The first IMF is marked on them, which is taken into account in further analysis of the signal. The signals presented in the graph represent a significant degree of damage, and their characteristic features are visible.

**Figure 7:**
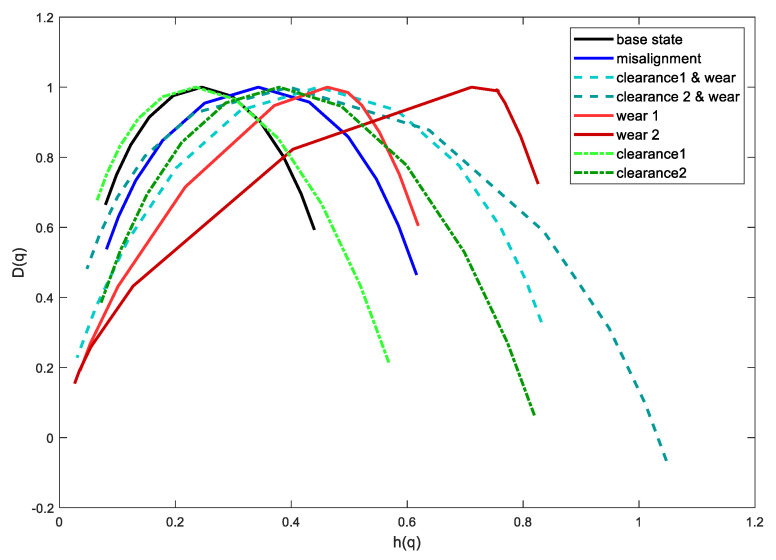
Multifractal spectra.

Twenty records for each condition were used for further analysis, each 1 s long (10,000 samples) for a total of 220 records. Each record was subjected to the EMD-WLMF analysis. Figure 7 shows the multifractal spectra for various operational states of the gears. The examples show up to two cases per state to increase the visibility of the image.

The least multifractal character is for the fault-free state. As the damage develops, the width of the spectrum increases. The spectral peak is also shifted. For the base state, the spectrum asymmetry index is close to zero. For the tested damages, the absolute value of the asymmetry index increases, and for the wear condition of the gears it has the opposite sign than for the increased backlash. For mixed states of damage, the spectrum is characterized by a large width and indices of asymmetry varying within wide limits.

**Figure 8:**
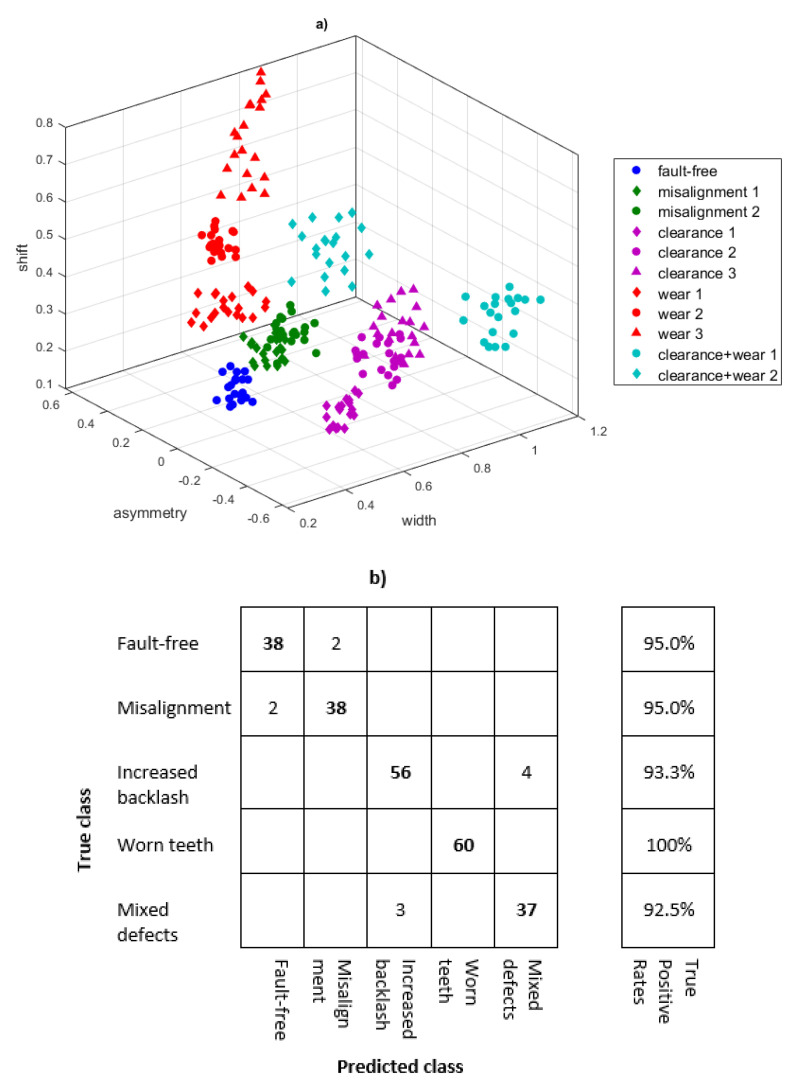
Classification of damage (**a**) scatter plot of three parameters of the multifractal spectrum, (**b**) confusion matrix for the test set performed with SVM method.

Three parameters were selected for the diagnosis of the gear condition: the width of the multifractal spectrum, the asymmetry index, and the singularity of the greatest dimension. The list of these parameters is presented in the scatter plot (Figure 8a).

As the damage develops, the level of multifractality of the signal increases and the spectrum is wider. The asymmetry index for the ‘base state’ is close to zero, and not much higher for the ‘misalignment’ state. The states of increased backlash and wear are characterized by the opposite sign of the spectrum asymmetry index and an increase in its absolute value. The spectrum shift indicator also changes for different operating states.

Any known machine learning method—e.g., k-nearest neighbours (k-NN), support vector machines (SVM), or neural network (NN)—can be used to classify the damage [25]. The fault classification was performed for a newly generated test set of data (240 records) with support vector machines (SVM). The confusion matrix is shown in Figure 8b. The average true positive rate for the test set is 95.16%. The faulty recognition of the state was for ‘fault-free’ and initial ‘misalignment’. The remaining states found to be in error are ‘increased backlash’ and ‘worn teeth and increased backlash’. The tests performed on the laboratory stand brought the desired effect. The influence of damage development on the parameters of the multifractal spectrum was presented. However, the stand lacks additional disturbances that accompany the operation of real machines.

### 3.2. Analysis of the Transmission Vibration Signal in a Car

**Figure 9:**
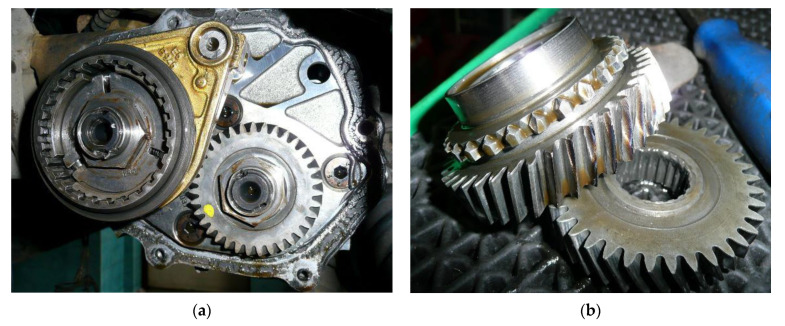
Test object (**a**) view of the gearbox, (**b**) pair of gear wheels with signs of wear.

The tests were carried out on a Punto five-speed gearbox (Figure 9a). The experiment was carried out during road tests for various rotational speeds and loads. The experiment used the recorded signals of acceleration of vibrations of the gearbox housing and additional signals from the crankshaft position and throttle position sensor.

The vibration accelerations of the gearbox housing were measured with a Bruel & Kjaer type IEPE No. 4514 sensor. The signals were recorded with a Bruel & Kjaer PULSE type 3560E portable data recorder with a sampling frequency of 65,536 Hz.

Signals of a duration of about 1 min were recorded while driving at the most preset speed possible. Additional signals enabled signal synchronization and measurement of rotational speed and load.

The active experiment consisted in registering signals under simulated mechanical damage to the gearbox, mimicking those often diagnosed in car repair shops. Such damage includes, among others, wear of the gearbox teeth.

In order to investigate the influence of gearbox teeth wear on the vibration signal, an active experiment was performed for the following conditions:

- gearbox in good condition
- fifth gear drive gear teeth worn at about one-third of the circumference
- fifth gear drive gear teeth heavily worn at about one-third of the circumference (Figure 9b)
- gearbox after replacing worn wheels with new ones.

**Figure 10:**
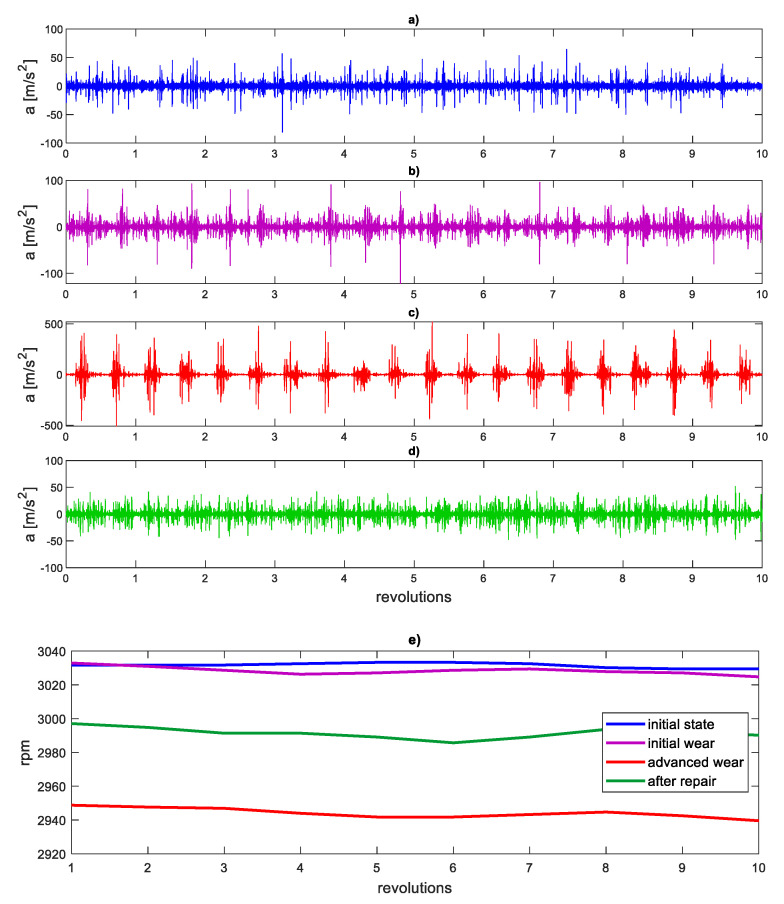
Time series of the first IMF for (**a**) initial state, (**b**) initial wear, (**c**) advanced wear, (**d**) after repair, (**e**) examples of rotational speed changes during registration.

Figure 10a–d show the first IMF waveforms of the gearbox housing vibration signal for four operating states, and Figure 10e shows examples of changes in rotational speed during the record registration.

**Figure 11:**
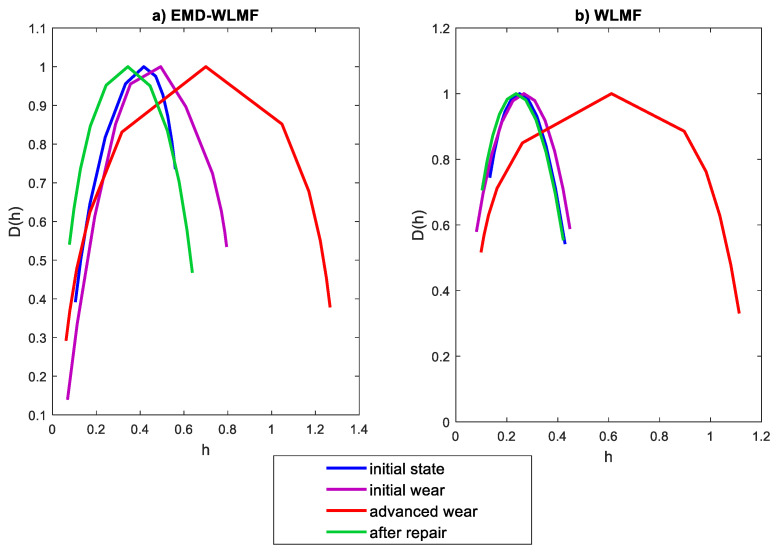
Sample multifractal spectra of (**a**) EMD-WLMF, (**b**) WLMF.

The EMD-WLMF algorithm was tested for 80 records, 20 records for each state. Figure 11a presents sample multifractal spectra for four states, and Figure 11b presents multifractal spectra for the same states using the only WLMF algorithm.

**Figure 12:**
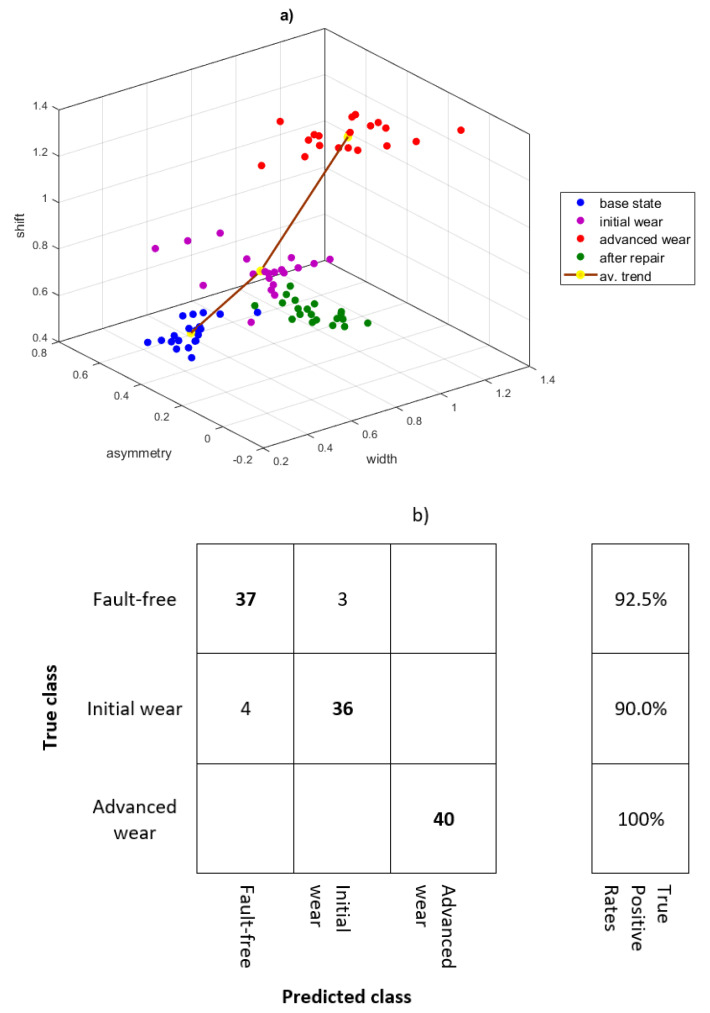
Damage classification based on three features of the multifractal spectrum (**a**) and confusion matrix (**b**).

By comparing the multifractal spectra for the first IMF of the vibration signal and the raw of the vibration signal, it can be concluded that the introduction of the EMD enables the identification of tooth wear at an early stage. In Figure 10b, the spectrum at initial wear almost does not differ from the base state and the spectrum for a new pair of gears. The EMD-WLMF analysis showed that the spectrum for the new pair of gears (‘after repair’) is the most symmetrical. The spectrum for the ‘initial state’ is narrower, but shows a shift and asymmetry. As the wear develops, the spectrum becomes wider, shifted along the h(q) axis, and shows asymmetry. The illustration of spectrum features for all 80 records is shown in Figure 12a.

The scatter plot for more records confirms that the spectrum width increases as the wear develops. The asymmetry coefficient for all states has a large spread, while for the new pair of teeth (‘after repair’) the spectrum is the most symmetrical. As the wear of the teeth increases, the spectrum shifts to the right. The average trend of damage development was marked. For a new pair of teeth (‘after repair’), the development of the damage may follow a ‘different path’ on the scatter plot, but the increasing trend of the width indicators and spectrum shift should be analogous.

The fault classification was performed for a newly generated test set of data (120 records) with support vector machines (SVM). The confusion matrix is shown in Figure 12b. The average true positive rate for the test set is 94.17%. The faulty recognition of the state was for ‘fault-free’ and ‘initial wear’. Due to the dispersion of the data, the diagnosis can be confirmed on the basis of the average trend shown in Figure 12a.

### 3.3. Analysis of the Vibration Signal of the Internal Combustion Engine Head

The next example shows the analysis of the vibration signal of the engine head signal recorded under operating conditions.

The tests were carried out on the drive system of the Fiat Punto car, with a four-cylinder 1.2 spark-ignition engine. The experiment was carried out during road tests for various rotational speeds and loads. The following dynamic signals were recorded:

- acceleration of vibrations of the cylinder head at the first cylinder in the vertical and horizontal directions
- acceleration of vibrations of the cylinder head at the fourth cylinder in the vertical direction

and additional signals

- from the crankshaft position sensor
- ignition in the first cylinder
- throttle position.

Vibration accelerations of the cylinder head were measured with Bruel & Kjaer DeltaShear type 4393 sensors, mounted with a threaded connection. The signals were recorded using a Bruel & Kjaer PULSE type 3560E portable data recorder with a sampling frequency of 65,536 Hz.

Signals of approximately 1 min duration were recorded while driving at a constant speed. Additional signals made it possible to identify engine cycles, injection and ignition timing, and valve timing.

**Figure 13:**
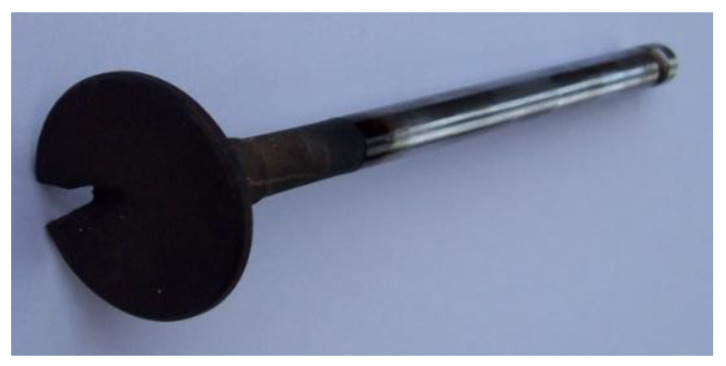
View of the damage of outlet valve.

A leak in the piston-cylinder system may be caused by burnout of the exhaust valve. During the tests, such damage was simulated by cutting the valve plug at a length of approx. 3 mm (defect 1) and 6 mm (defect 2)—Figure 13.

The process of generating vibrations in an internal combustion engine is very complex. The measured vibrations are a combination of periodic waves related to the operation of rotating elements and responses to impulse excitations related to reciprocating motion, as well as excitations caused by gas pressure. Strong transients in the vibroacoustic signal come from the work of the intake and exhaust valves, injectors, the combustion process, and piston strokes against the cylinder sleeve. Additional impulse excitation can be a response to mechanical failures not detected by the on-board diagnostic (OBD) system [49,50].

**Figure 14:**
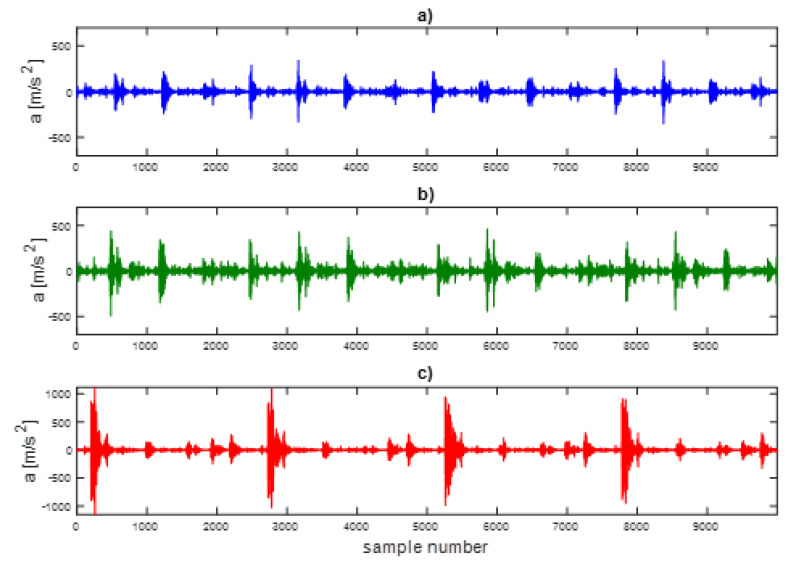
Acceleration time series of the engine head vibrations—the first IMF for (**a**) base state, (**b**) defect 1, (**c**) defect 2.

The time series of vibration accelerations after determining the first IMF for four engine operation cycles and three operating states are shown in Figure 14.

**Figure 15:**
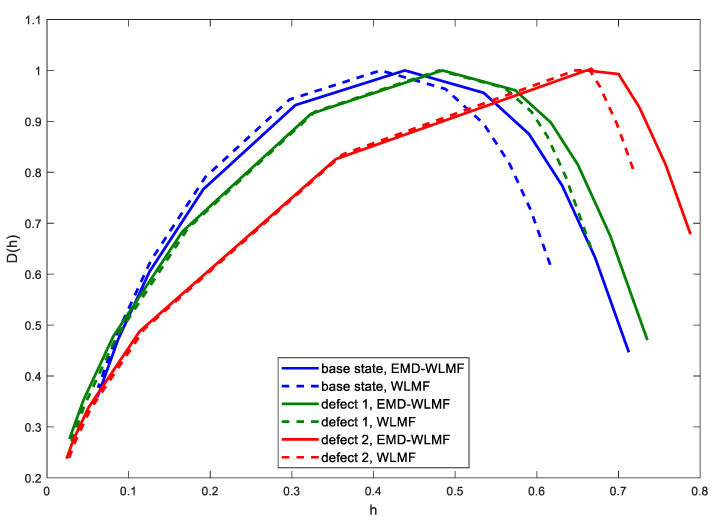
Multifractal spectrum of the vibration signal for three states of valves.

Figure 15 presents exemplary multifractal spectra for three operating states using the WLMF and EMD-WLMF algorithms. Due to the presence of transients in the signal, a large share of responses to impulse excitations—such as valve closing, ignition, piston hitting the cylinder, etc.—of the multifractal spectrum after EMD application does not differ much from the spectrum without EMD filtration. As a result of the damage development, the spectrum becomes wider and its maximum value for q = 0 is shifted to the right.

**Figure 16:**
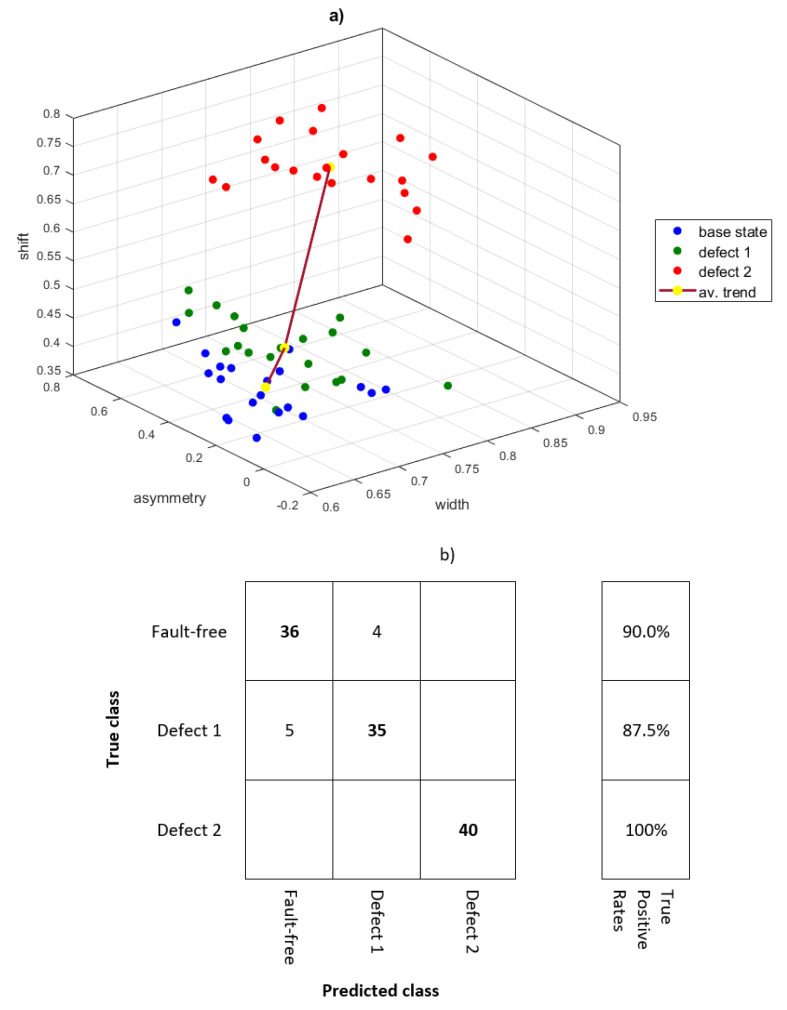
Damage classification based on three features of the multifractal spectrum (**a**) and confusion matrix (**b**).

Figure 16 shows the scatter plot for the three operational states, each with 20 records. As the damage develops, the values of the ‘width’ and ‘shift’ parameters increase. The ‘asymmetry’ parameter is characterized by the largest dispersion of values, although it also increases taking into account the average values for the entire set. The average trend for damage development is marked.

The fault classification was performed for a newly generated test set of data (120 records) with support vector machines (SVM). The confusion matrix is shown in Figure 16b. The average true positive rate for the test set is 92.5%. The faulty recognition of the state was for ‘fault-free’ and ‘defect 1’. Due to the dispersion of the data, the diagnosis can be confirmed on the basis of the average trend shown in Figure 16a.

## 4. Discussion

The article uses the method of wavelet leaders’ multifractal analysis dedicated to the first IMF of the vibration signal of rotating machines. The presented method can be applied to dynamic signals in non-stationary conditions due to the use of the EMD and wavelet analysis methods. Previous works [32] used the EMD analysis and the MF-DFA algorithm to diagnose the damage. However, the WLMF algorithm shows lower computational costs, numerical stability, and high versatility in terms of real signals compared to MF-DFA. The method corresponds to the need to find a relatively fast algorithm for diagnosing the condition of machines in non-stationary conditions. It is a statistical method. It can be an alternative to methods based on a set of signal features. Failure classification can be done using one of the machine learning methods, such as SVM, k-NN, or a neural network based on the features of the multifractal spectrum.

The method has been tested for signals recorded on a laboratory stand and for real objects. Damage was distinguished based on three features of the multifractal spectrum, named here: width, asymmetry, and shift. The tests performed on the laboratory stand brought a good effect. Multifractal analysis of only the first IMF allowed to filter out harmonics that do not show multifractality features. The development of damage to the gear transmission was reflected in the examined features of the multifractal spectrum. However, the stand lacks additional disturbances that accompany the operation of real machines.

The next two investigated cases are the diagnosis of mechanical damage to the vehicle while driving, such as gearbox wear and burnout of the exhaust valves of the internal combustion engine. The signals were recorded in non-stationary conditions. Due to the fluctuations in rotational speed and load, which could affect the dynamics of the signal, the dispersion of the features of the multifractal spectrum was much greater than that observed in the tests on the laboratory stand. Due to the dispersion of the features of the multifractal spectrum, the average value of these features can be used to diagnose the damage. By comparing the multifractal spectra for the first IMF of the vibration signal and the raw of the vibration signal of the gearbox, it can be concluded that the introduction of the EMD enables the identification of tooth wear at an early stage. Due to the presence of transients of the engine head vibrations signal, a large share of responses to impulse excitations—such as valve closing, ignition, piston hitting the cylinder, etc.—the multifractal spectrum after EMD application does not differ much from the spectrum without EMD filtration.

Like any method, this one also has its limitations. The main limitation is the multifractality of the signal, so the signal must contain impulse excitations caused by damage, as in a toothed gear. If the damage causes an increase in the harmonic component, the change in the multifractal spectrum will be hardly noticeable and then other methods—e.g., order tracking or the AR reference model—will bring better results.

## Data Availability

Not applicable.
